# Supplementary material for: Transcriptome Co-Expression Network Analysis of Peach Fruit with Different Sugar Concentrations Reveals Key Regulators in Sugar Metabolism Involved in Cold Tolerance
Source: Foods. 2023 Jun 1;12(11):2244. doi: 10.3390/foods12112244 (PMC10252351; doi:10.3390/foods12112244)
Supplement: Supplementary file 1 [file foods-12-02244-s001.zip › foods-2406598-supplementary.pdf]

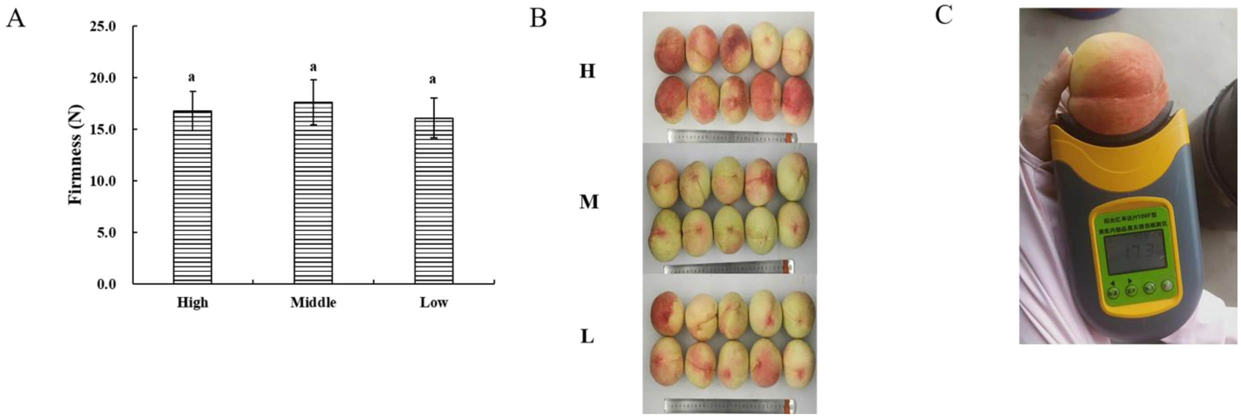

Figure S1. Physiological indexes of peach fruit at picking time. The firmness of high-sugar, moddle-sugar and low-sugar peach fruit at picking time (0 d) (A), the external color of peach fruit at 0 d (B) and TSS of peach fruit was measured by a detector during picking (C).

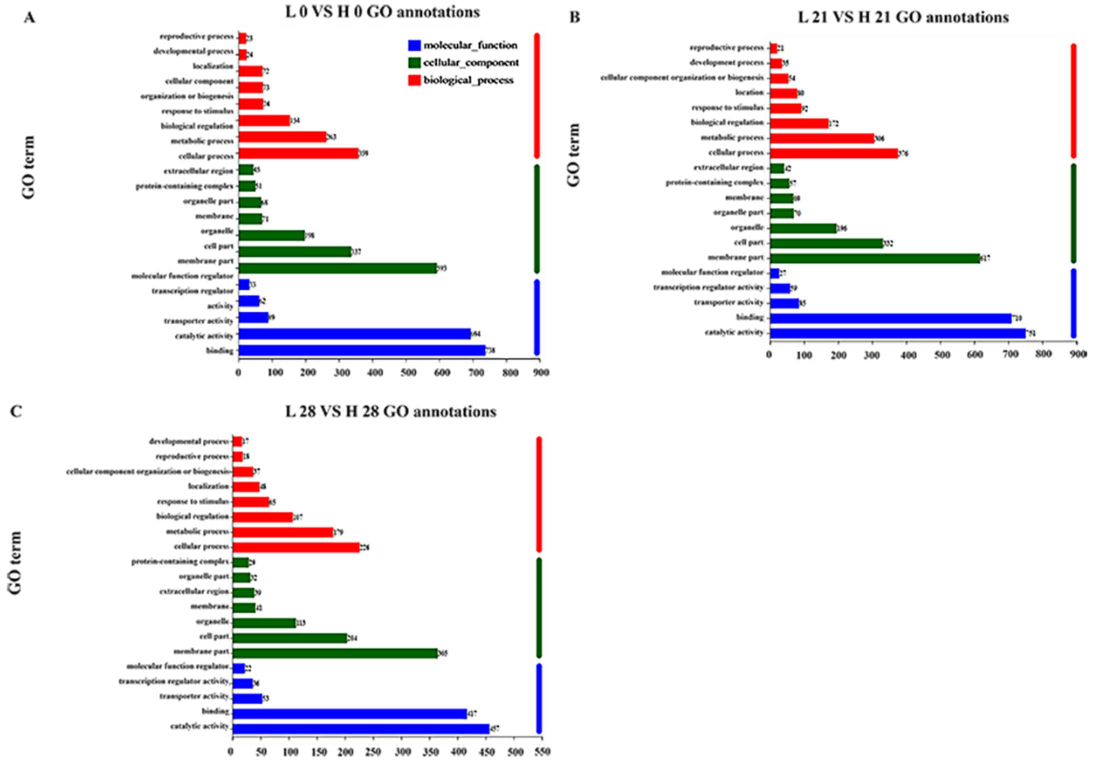

Figure S2. GO function of the DEGs in the three comparison groups of peach fruit. GO function classification of the DEGs in L 0 VS H 0 (A), GO function classification of the DEGs in L 21 VS H 21 (B), GO function classification of the DEGs in L 28 VS H 28 (C).

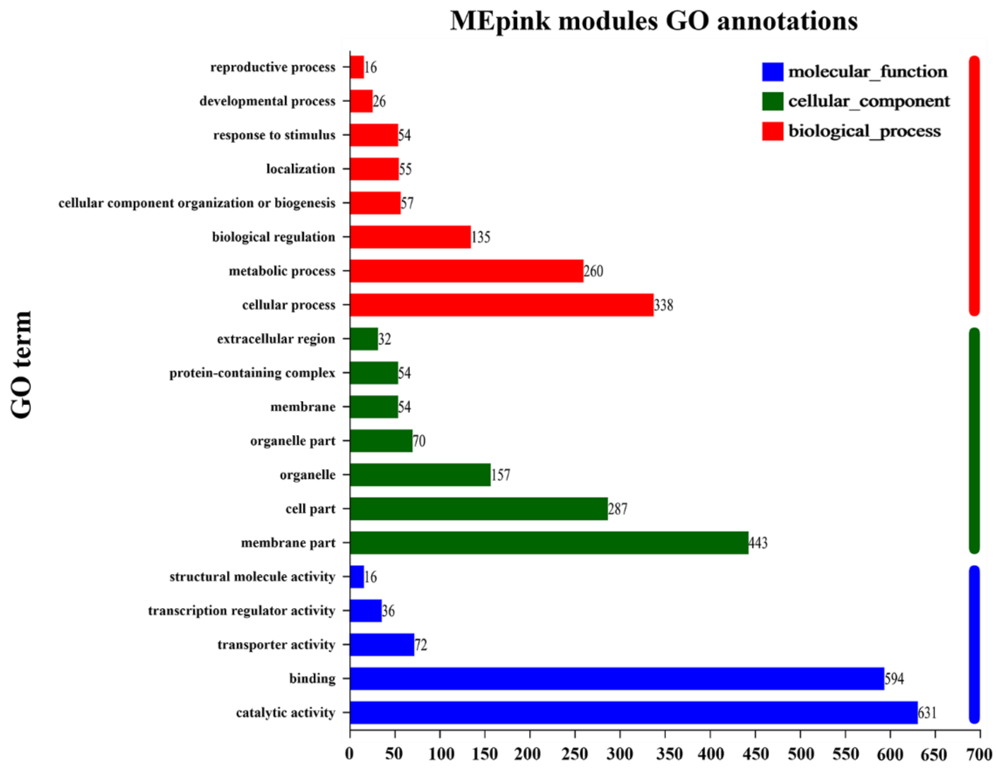

Figure S3. GO function classification of 1468 DEGs in MEpink module.

Supplementary Table S1.

Primers used for quantification of mRNA levels by qRT-PCR

Gene name	Accession no.	Sequence	Product (bp)
PpSS-FP	Prupe.5G241700	GGGCTGACCAATCTGTCTACT	102
PpSS-RP	Prupe.5G241700	CGTTTTCCGCCTTATTGTAGAG	
PpMGAM-FP	Prupe.4G103600	GGAGCTGCGAGGTAGGTGGTAAGCA	149
PpMGAM-RP	Prupe.4G103600	ATGTGAGGCGTGATAGGACTTTTTT	
PpINV-FP	Prupe.3G009500	TCAAGAAGGGATGGTCTGGACT	192
PpINV-RP	Prupe.3G009500	CATCTGCCTGTGCTGCTGTGAC	
PpHXK-FP	Prupe.3G057800	GTTTGACTACATTGCAGCAGAACT	208
PpHXK-RP	Prupe.3G057800	ACTCAATTCAGCCACAACATCTTG	
PpFRK-FP	Prupe.3G160500	GAAGAGTCAAGGGTATGAAGGTA	230
PpFRK-RP	Prupe.3G160500	TTTGAGCATGACATTCAGCACAG	
PpMYB1-FP	Prupe.6G229000	CGACAACGCCATCAAGAACCAC	102
PpMYB1-RP	Prupe.6G229000	TAGCCTCCGCTTCCACCTACAC	
PpMYB3-FP	Prupe.5G182000	TGGGAAATCTAACCTTGGGACA	149
PpMYB3-RP	Prupe.5G182000	GAGAGTTAGAGTCGACGTGTCCA	
PpWRKY4-FP	Prupe.8G265900	ACATTCAGTCCCCAATTAGATC	104
PpWRKY4-RP	Prupe.8G265900	CAAGAGGAACCCAAGAGTGCTA	
PpbHLH2-FP	Prupe.6G159200	AATTACCACAACGTAATCCTGC	151
PpbHLH2-RP	Prupe.6G159200	GACTCTGACATCTTCCCTTCCT	
PpbZIP1-FP	Prupe.7G160600	GGGTTCTTCTAGTGGGGTTTCT	116
PpbZIP1-RP	Prupe.7G160600	CCCGATTGGATAGCATTCTTTT	
PpbZIP2-FP	Prupe.8G091600	GATGATTTGATGGCTCAGATGG	234
PpbZIP2-RP	Prupe.8G091600	AGTGGGCTCATTGAAGCTGCTCGAA	
PpbZIP3-FP	Prupe.1G374400	AATTCCTCTGGTTCCACTCAGCTTC	216
PpbZIP3-RP	Prupe.1G374400	GGTCGTGATGTTTATGCTGGTC	
PpMYB-related1-FP	Prupe.2G176200	ATGGGCCTTCCGGGTCTCAATTCTA	180
PpMYB-related1-RP	Prupe.2G176200	CTCCCTGGACTTGGTAATCGTA	
PpTEF2-FP	Prupe.4G138900	GGTGTGACGATGAAGAGTGATG	129

PpTEF2-RP	Prupe.4G138900	TGAAGGAGAGGGAAGGTGAAAG

*Primers are indicated in the 5´→3´direction

Supplementary Table S2.

The quality and statistical summary of the transcriptome study data mapped to the peach genome

Sample	Raw reads	Clean reads	Error rate (%)	Q20 (%)	Q30 (%)	GC content (%)
H0_01	44994492	44551998	0.03	97.91	93.93	46.42
H0_02	43697494	43336662	0.03	97.97	94.11	46.56
H0_03	43606334	43094710	0.03	97.86	93.86	46.57
L0_01	43922994	43412198	0.02	98.09	94.40	46.60
L0_02	43471474	42975876	0.03	97.90	93.97	46.58
L0_03	43865902	43292868	0.03	97.82	93.77	46.71
H21_01	44226152	43677818	0.03	97.91	93.99	46.56
H21_02	43896830	43266204	0.03	97.95	94.10	46.61
H21_03	42163572	41538068	0.02	98.03	94.29	46.60
L21_01	43189442	42741282	0.03	97.93	94.04	46.49
L21_02	43842088	43316462	0.03	97.96	94.08	46.59
L21_03	43836170	43382696	0.03	97.88	93.94	46.57
H28_01	43316782	42780332	0.03	97.81	93.77	46.48
H28_02	44317212	43613994	0.03	97.94	94.09	46.72
H28_03	44473576	43788868	0.03	97.87	93.92	46.67
L28_01	43592032	42932950	0.03	97.85	93.88	46.56
L28_02	43501166	42973864	0.03	97.83	93.83	46.65
L28_03	43745074	43164166	0.03	97.81	93.76	46.51

Supplementary Table S3.

Pearson's correlation coefficients analysis among the transcriptome study samples

	H0_01	H0_02	H0_03	L0_01	L0_02	L0_03	H21_03	H21_01	H21_02	L21_02	L21_01	L21_03	H28_02	H28_01	H28_03	L28_01	L28_02	L28_03
H0_01	1.00	1.00	1.00	0.96	0.97	0.94	0.53	0.50	0.51	0.53	0.53	0.53	0.51	0.50	0.52	0.40	0.41	0.41
H0_02	1.00	1.00	1.00	0.96	0.97	0.96	0.51	0.48	0.49	0.52	0.51	0.51	0.49	0.48	0.50	0.39	0.40	0.39
H0_03	1.00	1.00	1.00	0.97	0.97	0.96	0.52	0.50	0.51	0.54	0.53	0.52	0.51	0.50	0.52	0.40	0.41	0.40
L0_01	0.96	0.96	0.97	1.00	0.98	0.99	0.51	0.49	0.50	0.54	0.52	0.51	0.49	0.48	0.50	0.39	0.39	0.39
L0_02	0.97	0.97	0.97	0.98	1.00	0.99	0.48	0.46	0.47	0.49	0.48	0.48	0.45	0.44	0.46	0.36	0.37	0.36
L0_03	0.94	0.96	0.96	0.99	0.99	1.00	0.46	0.44	0.45	0.48	0.47	0.46	0.43	0.42	0.44	0.34	0.35	0.34
H21_03	0.53	0.51	0.52	0.51	0.48	0.46	1.00	0.99	0.99	0.90	0.93	0.94	0.91	0.92	0.91	0.94	0.94	0.94
H21_01	0.50	0.48	0.50	0.49	0.46	0.44	0.99	1.00	0.99	0.89	0.91	0.92	0.89	0.89	0.89	0.95	0.94	0.94
H21_02	0.51	0.49	0.51	0.50	0.47	0.45	0.99	0.99	1.00	0.93	0.93	0.93	0.91	0.90	0.90	0.94	0.94	0.94
L21_02	0.53	0.52	0.54	0.54	0.49	0.48	0.90	0.89	0.93	1.00	0.98	0.97	0.93	0.92	0.92	0.86	0.87	0.88
L21_01	0.53	0.51	0.53	0.52	0.48	0.47	0.93	0.91	0.93	0.98	1.00	0.99	0.94	0.95	0.94	0.88	0.90	0.91
L21_03	0.53	0.51	0.52	0.51	0.48	0.46	0.94	0.92	0.93	0.97	0.99	1.00	0.94	0.95	0.95	0.89	0.91	0.92
H28_02	0.51	0.49	0.51	0.49	0.45	0.43	0.91	0.89	0.91	0.93	0.94	0.94	1.00	0.99	0.99	0.93	0.94	0.94
H28_01	0.50	0.48	0.50	0.48	0.44	0.42	0.92	0.89	0.90	0.92	0.95	0.95	0.99	1.00	0.99	0.93	0.94	0.94
H28_03	0.52	0.50	0.52	0.50	0.46	0.44	0.91	0.89	0.90	0.92	0.94	0.95	0.99	0.99	1.00	0.92	0.93	0.93
L28_01	0.40	0.39	0.40	0.39	0.36	0.34	0.94	0.95	0.94	0.86	0.88	0.89	0.93	0.93	0.92	1.00	1.00	0.99
L28_02	0.41	0.40	0.41	0.39	0.37	0.35	0.94	0.94	0.94	0.87	0.90	0.91	0.94	0.94	0.93	1.00	1.00	1.00
L28_03	0.41	0.39	0.40	0.39	0.36	0.34	0.94	0.94	0.94	0.88	0.91	0.92	0.94	0.94	0.93	0.99	1.00	1.00

Supplementary Table S4.

The information of regulatory network of related gene

Gene name	Accession no.	Gene symbol	Gene description
PpSS	Prupe.5G241700	LOC18777229	sucrose synthase 6
PpMGAM	Prupe.4G103600	LOC18781275	alpha-glucosidase
PpINV	Prupe.3G009500	LOC18788064	insoluble isoenzyme CWINV1
PpHXK	Prupe.3G057800	LOC18781930	hexokinase-1
PpFRK	Prupe.3G160500	LOC18783524	fructokinase-6
PpERF1	Prupe.2G272500	LOC18785268	ethylene-responsive transcription factor ERF106
PpERF2	Prupe.2G289500	LOC18787317	dehydration-responsive element-binding protein 1A
PpERF3	Prupe.1G130300	LOC18789727	ethylene-responsive transcription factor RAP2-3
PpERF4	Prupe.4G176200	LOC18778290	ethylene-responsive transcription factor 4

PpERF5	Prupe.5G090100	LOC18776409	dehydration-responsive element-binding protein 1E
PpERF6	Prupe.6G039700	LOC18772665	pathogenesis-related genes transcriptional activator PTI6
PpERF7	Prupe.6G064700	LOC18772983	chromatin modification-related protein eaf-1
PpERF8	Prupe.7G194400	LOC18771728	ethylene-responsive transcription factor ERF017
PpHB-other1	Prupe.1G271400	LOC18792206	homeobox-leucine zipper protein ATHB-13
PpHB-other2	Prupe.2G156900	LOC18787087	homeobox-leucine zipper protein ANTHOCYANINLESS 2
PpHB-other3	Prupe.2G241700	LOC18784831	BEL1-like homeodomain protein 9
PpHB-other4	Prupe.6G102300	LOC18773641	homeobox-leucine zipper protein REVOLUTA
PpHB-other5	Prupe.6G263900	LOC18772447	BEL1-like homeodomain protein 9
PpHB-other6	Prupe.7G079300	LOC18771696	homeobox-leucine zipper protein HOX11
PpHB-other7	Prupe.7G149700	LOC18769821	homeobox-leucine zipper protein ATHB-40
PpMYB1	Prupe.6G229000	LOC18775054	transcription factor MYB44
PpMYB2	Prupe.1G323400		Myblike DNAbinding domain containing protein
PpMYB3	Prupe.5G182000	LOC18777189	transcription factor MYB1R1
PpMYB4	Prupe.4G035100	LOC18781079	transcription repressor MYB6
PpMYB5	Prupe.6G267600	LOC18772814	transcription factor MYB1
PpMYB6	Prupe.6G303300	LOC18772237	transcription factor MYB86
PpMYB7	Prupe.6G085000	LOC18773813	transcription factor MYB59
PpNAC1	Prupe.1G383200	LOC18791355	NAC domain-containing protein 79
PpNAC2	Prupe.2G202000	LOC18787361	NAC domain-containing protein 67
PpNAC3	Prupe.2G202600	LOC109947178	NAC domain-containing protein 100-like
PpNAC4	Prupe.3G014400	LOC18782146	NAC domain-containing protein 86
PpNAC5	Prupe.4G143600	LOC18780262	NAC domain-containing protein 21/22
PpNAC6	Prupe.7G217000	LOC18771576	protein BEARSKIN2
PpWRKY1	Prupe.1G071400	LOC18789894	probable WRKY transcription factor 11
PpWRKY2	Prupe.1G114800	LOC18789015	probable WRKY transcription factor 48
PpWRKY3	Prupe.3G214800	LOC18782434	probable WRKY transcription factor 31
PpWRKY4	Prupe.8G265900	LOC18766660	WRKY transcription factor 69
PpWRKY5	Prupe.6G286000	LOC18772845	probable WRKY transcription factor 33
PpbHLH1	Prupe.1G304900	LOC18793925	transcription factor bHLH30
PpbHLH2	Prupe.6G159200	LOC18775069	transcription factor HEC2
PpbHLH3	Prupe.3G312800	LOC18782429	transcription factor ICE1
PpbHLH4	Prupe.7G071500	LOC18770320	transcription factor bHLH130

PpbHLH5	Prupe.8G242100	LOC18767836	basic helix-loop-helix protein A
PpbZIP1	Prupe.7G160600	LOC18769167	bZIP transcription factor 44
PpbZIP2	Prupe.8G091600	LOC18767767	bZIP transcription factor 11
PpbZIP3	Prupe.1G374400	LOC18788985	bZIP transcription factor 11
PpbZIP4	Prupe.6G343100	LOC18773097	light-inducible protein CPRF2
PpARF1	Prupe.2G190400	LOC18786825	auxin response factor 18
PpARF2	Prupe.6G097700	LOC18772269	auxin response factor 4
PpARF3	Prupe.8G252300	LOC18768867	auxin response factor 2
PpHSF1	Prupe.1G021200	LOC18790318	heat stress transcription factor A-6b
PpHSF2	Prupe.1G433500	LOC18788483	heat stress transcription factor B-4
PpHSF3	Prupe.4G046000	LOC18779732	heat stress transcription factor B-2a
PpMYB_related1	Prupe.2G176200	LOC18784574	protein REVEILLE 3
PpMYB_related2	Prupe.6G106200	LOC18772343	
PpDof1	Prupe.1G388600	LOC109946797	dof zinc finger protein DOF3.4
PpDof2	Prupe.5G173100	LOC18776482	dof zinc finger protein DOF5.6
PpGRAS	Prupe.5G091700	LOC18776291	scarecrow-like protein 28
PpTCP	Prupe.4G171100	LOC18780672	transcription factor TCP4
PpLBD (AS2/LOB)	Prupe.4G105700	LOC18781496	LOB domain-containing protein 4
PpLBD (AS2/LOB)	Prupe.3G290800	LOC18781820	LOB domain-containing protein 25
PpSBP	Prupe.4G050400	LOC18778248	squamosa promoter-binding-like protein 3
PpSBP	Prupe.1G290200	LOC18790408	squamosa promoter-binding-like protein 6
PpSBP	Prupe.7G074200	LOC18769457	squamosa promoter-binding-like protein 9
PpDBB	Prupe.3G155900	LOC18784147	B-box zinc finger protein 24
PpMIKC	Prupe.1G549600	LOC18789032	agamous-like MADS-box protein AGL11
PpMIKC	Prupe.3G170600	LOC18783633	agamous-like MADS-box protein AGL1
PpB3	Prupe.4G049500	LOC18779046	B3 domain-containing transcription factor LEC2
PpAP2	Prupe.1G084900	LOC18791311	AP2-like ethylene-responsive transcription factor AIL5
PpNF-YA	Prupe.3G043900	LOC18782250	nuclear transcription factor Y subunit A-3
PpCPP	Prupe.1G019100	LOC18789798	CRC domain-containing protein TSO1
PpGRAS	Prupe.7G236100	LOC18769184	DELLA protein DWARF8
PpM_type2	Prupe.7G164100	LOC18769016	floral homeotic protein DEFICIENS
PpZF-HD	Prupe.7G155700	LOC18769526	zinc-finger homeodomain protein 6
PpAP23	Prupe.6G203000	LOC18773090	AP2-like ethylene-responsive transcription factor AIL6
